# Associations between cardiovascular health and female infertility: A national population-based study

**DOI:** 10.1371/journal.pone.0306476

**Published:** 2024-07-05

**Authors:** Meiyan Luo, Jianshu Li, Xiangjun Xiao, Ping Wu, Ya Zhang

**Affiliations:** 1 Department of Obstetrics, The Affiliated Second Hospital, Hengyang Medical School, University of South China, Hengyang, China; 2 Nuclear Industry Health School, The Affiliated Nanhua Hospital, Hengyang Medical School, University of South China, Hengyang, China; 3 Department of Hand & Foot Surgery, The Affiliated Nanhua Hospital, Hengyang Medical School, University of South China, Hengyang, China; 4 Department of Nephrology, The Affiliated Nanhua Hospital, Hengyang Medical School, University of South China, Hengyang, China; 5 Department of Gland Surgery, The Affiliated Nanhua Hospital, Hengyang Medical School, University of South China, Hengyang, China; Albert Einstein College of Medicine, UNITED STATES

## Abstract

**Objective:**

This study investigates the relationship between cardiovascular health (CVH), as quantified by the American Heart Association’s Life’s Essential 8 (LE8) metric, and female infertility, utilizing data from the National Health and Nutrition Examination Survey (NHANES) spanning 2013–2018.

**Methods:**

We encompassed females aged 20–49 years and above from the NHANES in this cross-sectional analysis. We assessed CVH using the LE8 score, encompassing eight domains: dietary pattern, physical activity, nicotine exposure, sleep duration, body mass index (BMI), lipid profile, fasting blood glucose, and blood pressure levels. Logistic regression models were applied to explore the association between CVH scores and reported infertility, adjusting for potential confounders including age, race/ethnicity, and socioeconomic status.

**Results:**

Findings revealed a notable inverse association between CVH scores (per 10 scores) and female infertility [OR = 0.93, 95%CI: 0.90–0.96], Participants with higher CVH levels were 41% less likely to had female infertility compared to those with lower levels [OR = 0.59, 95%CI: 0.41–0.84]. Higher overall CVH scores, particularly in physical activity, BMI, and blood glucose, were associated with lower odds of infertility. This trend remained consistent across various demographic subgroups.

**Conclusion:**

Our findings underscore the significance of maintaining optimal cardiovascular health, as evidenced by higher LE8 scores, in mitigating the risk of female infertility. These insights advocate for the integration of CVH improvement strategies within the broader framework of reproductive health care, emphasizing the dual benefits of cardiovascular and reproductive health optimization.

## 1. Introduction

Female infertility, affecting an estimated 8–12% of couples globally, presents a complex interplay of factors involving both partners. While male factors contribute to approximately 50% of infertility cases, female factors alone are responsible for 20–30% [1, 2]. Studies have underscored the impact of early-life conditions on reproductive health, with a higher ponderal index at birth being associated with increased risk of infertility in female, suggesting the significance of in-utero development on long-term fertility outcomes [3]. Furthermore, secondary infertility is prevalent worldwide, often linked to infections and reproductive tract issues. The probability of conception decreases with age and duration of non-conception, with a notable decline in female fertility beginning around 25–30 years of age, intensifying after 35 [4].

Emerging evidence links female infertility with cardiovascular diseases (CVD), suggesting that female with infertility may have an increased risk of CVD later in life [5, 6]. For instance, factors like polycystic ovary syndrome (PCOS) and endometriosis, which are associated with infertility, have been observed to correlate with higher rates of CVD risk factors such as hypertension and metabolic syndrome [7]. Elevated levels of triglycerides and lower levels of high-density lipoprotein, markers of cardiovascular risk, have been observed in female with unexplained infertility, indicating a potential for atherogenic lipid profiles in this group [8]. Additionally, female infertility may act as a biomarker for future health risks, with reproductive health intricately tied to overall and long-term health outcomes [9].

The relationship between PCOS and cardiovascular risk illustrates the nuanced nature of this association. While PCOS is commonly associated with metabolic syndrome—a known CVD risk factor—the direct link to primary cardiovascular events such as stroke or myocardial infarction is more speculative. Studies provide mixed results, with some indicating a slight increase in cardiovascular events among female with menstrual irregularity, yet little evidence supports a strong association between hyperparathyroidism and cardiovascular events [10, 11]. This complexity underscores the importance of considering a wide array of reproductive health factors when assessing cardiovascular risk in female.

The American Heart Association’s Life’s Essential 8 (LE8) score encompasses a broad spectrum of cardiovascular health metrics, from diet and physical activity to blood pressure and blood glucose levels [12, 13]. Understanding how each component of the LE8 score relates to female infertility can provide insights into potential preventative strategies and therapeutic interventions [14].

The primary objective of this study is to elucidate the relationship between cardiovascular health (CVH), as gauged by the LE8 score, and female infertility, leveraging the comprehensive data from the National Health and Nutrition Examination Survey (NHANES).

## 2. Methods

### 2.1 Study population

**Fig 1:**
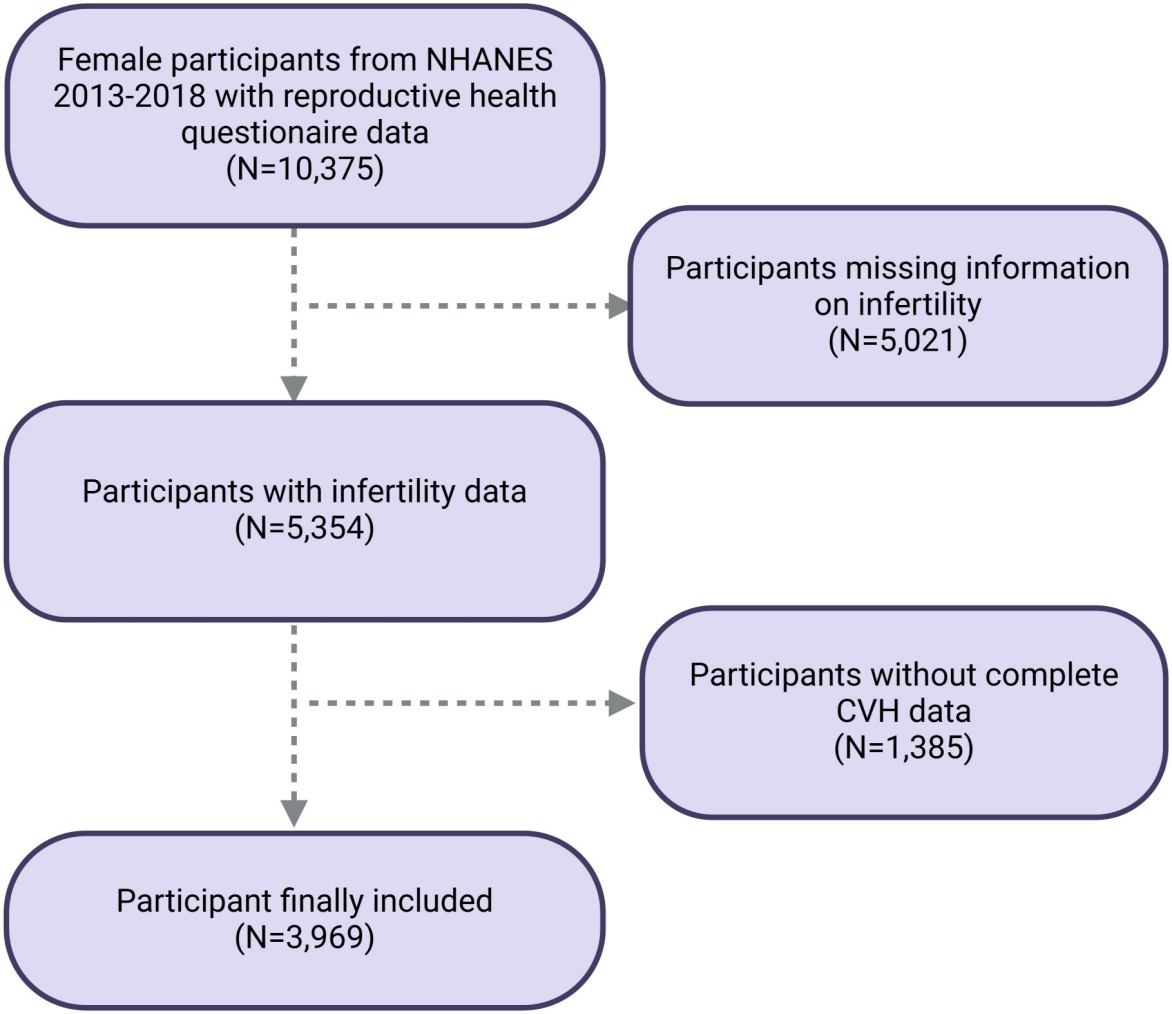
Flow chart of participant’s selection. NHANES, National Health and Nutrition Examination Survey.

Our study is based on an original cross-sectional analysis of the NHANES database, which serves as the setting for our research. The NHANES, conducted by the National Center for Health Statistics, is a pivotal program that aims to assess the health and nutritional status of the U.S. population [15, 16]. It provides critical data on the prevalence of major diseases and associated risk factors through extensive data collection. NHANES employs sophisticated multi-stage, probability-based sampling methods to ensure a nationally representative sample [17]. The program’s protocols have received approval from the National Center for Health Statistics (NCHS) Research Ethics Review Board, and all participants provided written informed consent by NHANES project team. This study utilized data from the 2013–2018 NHANES cycle. The study focused on female participants aged 20 years and older. From the initial pool of 10,375 females in the 2013–2018 cycle with reproductive questionnaire, the study excluded those with missing infertility data (n = 5,021), along with participants with missing or incomplete CVH data (n = 1,465), resulting in a final sample of 3,969 female participants for analysis (Fig 1).

### 2.2 Definition of infertility, CVH, and covariables

Infertility status was assessed based on responses to two queries (RHQ074 and RHQ076) within the Reproductive Health Questionnaire. RHQ074 inquired if participants had tried to conceive for a year or more without success, and RHQ076 asked if they had ever sought medical advice for difficulties in becoming pregnant [18, 19]. Affirmative responses to either question classified participants as having infertility. Cardiovascular health is evaluated through the LE8 score, which includes diet (aligned with the Dietary Approaches to Stop Hypertension (DASH) guidelines), physical activity, tobacco use, sleep, body mass index (BMI), lipid levels excluding high-density lipoprotein (HDL), blood glucose, and blood pressure. Standard surveys collect lifestyle data, while physical and biochemical measurements are taken following standardized methods. BMI is calculated as weight in kilograms divided by height in meters squared. Blood pressure is averaged from initial readings, non-HDL cholesterol is obtained by subtracting HDL from total cholesterol, and glycated hemoglobin is determined by liquid chromatography (detailed calculation see S1 Table). The LE8 score ranges from 0 to 100, with an aggregate score derived from these components, categorizing CVH into high, moderate, or low according to American Heart Association benchmarks [20, 21]. The selection of covariates for this study was guided by their potential influence on the connections between infertility and CVH. The covariates included age, race, education level, family income-to-poverty ratio (PIR), age of menarche and pelvic infection disease. The PIR is divided into three categories: low (PIR ≤1.3), medium (PIR > 1.3–3.5) and high (PIR > 3.5) [22]. These covariates were chosen based on their established relevance in epidemiological research and their potential confounding effects on the study outcomes [13].

### 2.3 Statistical analysis

The LE 8 score was used to stratify cardiovascular health into three categories: low (scores below 50), intermediate (scores between 50 and 79), and high (scores 80 or above). Percentage distributions were used for categorical data, while continuous data were expressed in terms of means and standard deviations. To compensate for any incomplete data, we utilized the mice package to perform multiple imputation using chained equations with ten imputation sets. The relationship between cardiovascular health status and infertility was investigated through a series of logistic regression models. The initial model did not adjust for additional variables; the second model accounted for age and ethnicity, and the third model further incorporated educational background, PIR, age of menstruation and history of pelvic inflammatory disease. Adjustment for PIR is a continuous variable in all models. Tests for linear trends across three categories of cardiovascular health metrics scores were performed by modeling the median value within each category as a continuous variable. To explore potential non-linear relationships between CVH and infertility, we employed smooth curve fitting techniques using generalized additive models (GAMs). GAMs allow for the flexible modeling of relationships between a predictor and an outcome variable by fitting a smooth curve instead of assuming a linear relationship. This approach is particularly useful for identifying and illustrating complex patterns in the data that might not be evident under linear analysis. The smoothness of the curve is determined by a smoothing parameter, which controls the trade-off between the model’s fit and its complexity. We assessed the appropriateness of the curve using the generalized cross-validation score as a criterion for selecting the smoothing parameter [23–25]. Interaction tests were conducted to assess whether the association between CVH and infertility differed across various subgroups, such as age, ethnicity, and socioeconomic status. For this purpose, we introduced interaction terms into our logistic regression models, which represent the product of CVH scores and subgroup identifiers. In addition, the same sensitivity analyses were conducted by restricting the age to the conventional age range for fertility (less than 50 years) to test the robustness of the results. All analyses were performed using the statistical software R, version 4.3.0, with a predetermined significance threshold set at a *P*-value of less than 0.05 for all two-tailed tests.

## 3. Results

### 3.1 Baseline characteristics

A total of 3,969 female participants with a mean age of 39.97 ± 11.42 years were included in this analysis, of which 476 (11.99%) were diagnosed with infertility. The mean LE8 score with its standard deviation (SD) was 66.76 (11.32). Of note, 16.00% had poor CVH (LE8 <50), 60.07% had moderate CVH (50 ≤ LE8 <80), and 23.94% were classified as having high CVH (LE8≥80). Participants with higher CVH scores had a lower prevalence of infertility compared to those with lower CVH scores (10.11% vs. 15.28%). In addition, participants with higher CVH scores were more likely to be younger and have older menarche, have high proportion of married or living with partner, have higher socioeconomic status (level of education and family income), and have a lower prevalence of diabetes mellitus and pelvic infection disease (P < 0.01) (Table 1). Characteristics of the participants aged 20–49 years are presented in S2 Table, and the results are generally consistent.

**Table 1:** Baseline characteristics of participants with CVH categories from LE8 score.

Characteristics	Low (LE8 <50)	Moderate(50≤ LE8 <80)	High (LE8 ≥80)	*P*-value
No. of participants in sample	635	2,384	950	
Age, y (SD)	45.26 ± 10.32	40.13 ± 11.33	36.03 ± 10.84	<0.001
Age of menarche, y (SD)	12.30 ± 1.84	12.55 ± 1.78	12.92 ± 1.75	<0.001
PIR	1.75 ± 1.33	2.41 ± 1.60	3.21 ± 1.66	
Race/ethnicity, n (%)				<0.001
Mexican American	89 (14.02)	423 (17.74)	121 (12.74)	
Others	48 (7.56)	250 (10.49)	112 (11.79)	
Non-Hispanic White	272 (42.83)	781 (32.76)	394 (41.47)	
Non-Hispanic Black	174 (27.40)	567 (23.78)	97 (10.21)	
Other Hispanic	52 (8.19)	363 (15.23)	226 (23.79)	
Marital status, %				<0.001
Married/Living with partner	284 (44.72)	1,177 (49.37)	521 (54.85)	
Living alone	351 (55.28)	1,207 (50.63)	429 (45.15)	
Education level, %				<0.001
Less than high school	162 (25.49)	358 (15.03)	55 (5.83)	
High school	203 (32.02)	577 (24.22)	112 (11.77)	
More than high school	270 (42.49)	1,449 (60.75)	783 (82.40)	
Diabetes, %				<0.001
Yes	225 (35.44)	295 (12.39)	28 (2.94)	
No	410 (64.56)	2,089 (87.61)	922 (97.06)	
Pelvic infection disease, %				<0.001
Yes	65 (10.24)	143 (6.00)	25 (2.63)	
No	570 (89.76)	2,241 (94.00)	925 (97.37)	
Infertility, %				0.008
Yes	97 (15.28)	283 (11.87)	96 (10.11)	
No	538 (84.72)	2,101 (88.13)	854 (89.81)	
AHA LE8 score (SD)				
Mean total CVH score	41.31 ± 6.78	65.18 ± 8.20	87.86 ± 5.40	<0.001
Mean DASH diet score	23.57 ± 26.14	38.62 ± 30.89	65.18 ± 28.87	<0.001
Mean physical activity score	9.54 ± 26.46	41.27 ± 45.89	88.59 ± 27.32	<0.001
Mean tobacco/nicotine exposure score	41.05 ± 40.23	72.46 ± 37.24	91.71 ± 19.65	<0.001
Mean sleep health score	67.20 ± 29.84	82.61 ± 24.53	91.39 ± 16.65	<0.001
Mean body mass index score	25.53 ± 28.31	52.04 ± 35.36	85.48 ± 23.53	<0.001
Mean blood lipid score	49.76 ± 31.46	72.05 ± 28.95	89.20 ± 20.16	<0.001
Mean blood glucose score	63.57 ± 31.66	86.17 ± 22.83	97.98 ± 8.91	<0.001
Mean blood pressure score	50.28 ± 30.62	76.21 ± 28.21	93.35 ± 15.46	<0.001

Mean (SD) for continuous variables: the P value was calculated by the weighted linear regression model.

Percentages for categorical variables: the P value was calculated by the weighted chi-square test.

Cardiovascular health (CVH) is categorized into 3 grades, low: LE8 score <50, mederate:50≤LE8 score <80, high: LE8 score ≥80.

Abbreviation: AHA, American Heart Association; LE8, Life’s Essential 8; CVH, cardiovascular health; DASH, Dietary Approaches to Stop Hypertension; PIR, The ration of family income to poverty.

### 3.2 Association between CVH and infertility

**Fig 2:**
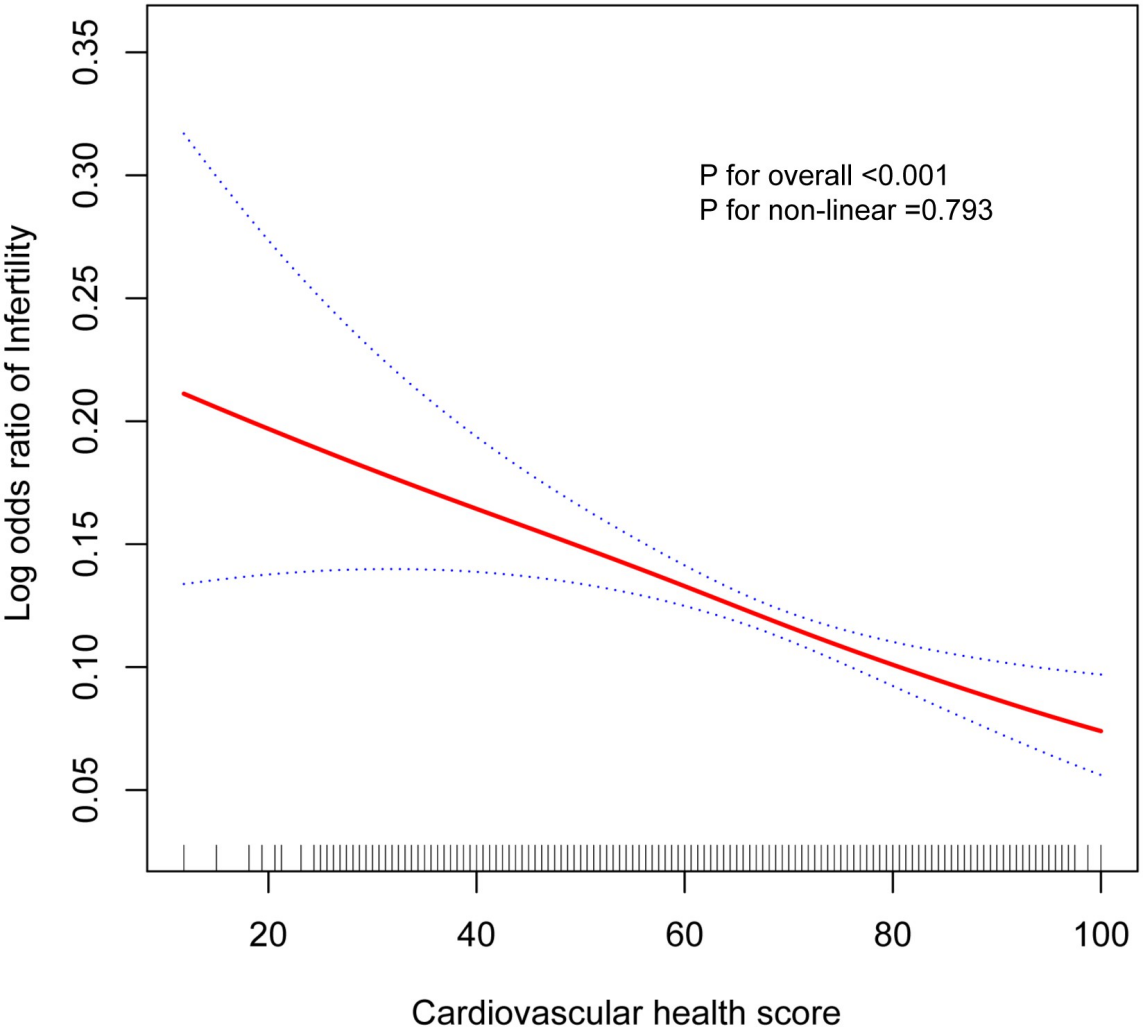
The association between total CVH score and infertility. The solid red line represents the smooth curve fit between variables. Blue bands represent the 95% of confidence interval from the fit.

Table 2 demonstrates the association of CVH score and its 8 subscores with infertility. In all models, there was an inverse association between total CVH score and infertility, with each 10-point increase in CVH associated with 7% lower odds of infertility after adjusting for all covariates [OR = 0.93, 95% CI: 0.90–0.96]. Participants with moderate CVH [OR = 0.76, 95% CI: 0.58–1.00] and higher CVH levels [OR = 0.59, 95% CI: 0.41–0.84] had 24% and 41% lower odds of infertility compared with the low CVH group, and there was a significant linear trend (*P* for trend < 0.01). For the association of the 8 subscores with infertility, also in the fully adjusted model, our results showed that it was mainly 3 of the subscores (physical activity score, body mass index score, and blood glucose score) that maintained a statistically significant negative association with infertility (*P* < 0.05). Associations for participants aged 20–49 years are presented in S3 Table, with generally consistent results. The dose-response relationship between LE8 score and infertility was assessed using smooth curve fitting. A negative association between LE8 score and infertility was observed in the fully adjusted model, but the non-linear relationship was not significant (total P value < 0.001, non-linear P-value = 0.793) (Fig 2).

**Table 2:** The associations between the Life’s Essential 8 cardiovascular health (CVH) score and infertility.

LE8/CVH	Model 1 [OR (95% CI)]	Model 2 [OR (95% CI)]	Model 3 [OR (95% CI)]
**Total CVH score (per 10 scores)**	0.87 (0.83, 0.91)	0.89 (0.85, 0.93)	0.93 (0.90, 0.96)
CVH categories			
Low (LE8 <50)	Ref.	Ref.	Ref.
Moderate (50≤ LE8 <80)	0.75 (0.58, 0.96)	0.82 (0.63, 1.06)	0.76 (0.58, 1.00)
High (LE8 ≥80)	0.62 (0.46, 0.84)	0.70 (0.51, 0.96)	0.59 (0.41, 0.84)
P for trend	<0.001	<0.001	<0.001
**Subclass CVH scores (per 10 socres)**			
Mean DASH diet score	0.96 (0.91, 1.01)	0.97 (0.92, 1.02)	0.97 (0.92, 1.01)
Mean physical activity score	0.95 (0.90, 1.00)	0.95 (0.89, 1.00)	0.95 (0.91, 0.99)
Mean tobacco/nicotine exposure score	0.92 (0.83, 1.01)	0.93 (0.83, 1.02)	0.93 (0.83, 1.02)
Mean sleep health score	0.96 (0.92, 1.01)	0.97 (0.93, 1.01)	0.97 (0.93, 1.02)
Mean body mass index score	0.93 (0.87, 0.98)	0.94 (0.89, 0.99)	0.94 (0.89, 0.99)
Mean blood lipid score	0.97 (0.93, 1.01)	0.98 (0.94, 1.02)	0.98 (0.94, 1.02)
Mean blood glucose score	0.93 (0.88, 0.98)	0.92 (0.87, 0.99)	0.92 (0.87, 0.99)
Mean blood pressure score	0.96 (0.91, 1.01)	0.96 (0.90, 1.02)	0.96 (0.91, 1.02)

Model 1 was unadjusted for covariates; Model 2 enhanced Model 1 by including age and ethnicity; and Model 3 further augmented Model 2 by integrating education level, family income-to-poverty ratio, marital status, age of menarche and pelvic infection disease. Abbreviation: CVH, cardiovascular health; DASH, Dietary Approaches to Stop Hypertension.

*Tests for linear trends across three categories of cardiovascular health metrics scores were performed by modeling the median value within each category as a continuous variable

#### 3.2.1 Subgroup analyses

Subgroup analyses based on different groups further demonstrated the robustness of the association between CVH and infertility across populations (Table 3). The results showed that the association between CVH and infertility was generally consistent across participants of different age, marital status, education, PIR, and diabetes status, all of which were inversely associated and not significantly different (all *P* for interaction > 0.05). The results of the subgroup analyses for participants aged 20–49 years are presented in S4 Table.

**Table 3:** Subgroup analysis of the associations of Life’s Essential 8 cardiovascular health (CVH) score (per 10 scores) and infertility.

Subgroup	OR (95%CI)	P for interaction
**Age**		0.205
< 40 years	0.88 (0.81, 0.95)	
≥ 40 years	0.92 (0.85, 1.00)	
**Marital status**		0.432
Married/Living with partner	0.95 (0.90, 1.00)	
Living alone	0.91 (0.86, 0.96)	
**PIR**		0.287
< 1.3	0.89 (0.79, 0.99)	
1.3–3.5	0.94 (0.88, 1.00)	
>3.5	0.93 (0.88, 1.01)	
**Education level**		0.540
Less than high school	0.84 (0.74, 0.94)	
High school	0.90 (0.83, 0.97)	
More than high school	0.91 (0.82, 0.99)	
**Diabetes**		0.391
Yes	0.85 (0.74, 0.96)	
No	0.93 (0.90, 0.97)	

Age, race, education level, family income-to-poverty ratio, marital status, age of menarche and pelvic infection disease were adjusted. Abbreviation: CVH, cardiovascular health.

## 4. Discussion

Our study involving 3,969 representative female participants showed associations between a comprehensive cardiovascular health indicator (CVH scores) and female infertility. Our results suggest that higher CVH levels are associated with a lower prevalence of infertility, especially significant in the CVH subscores corresponding to the three metrics of physical activity, BMI, and blood glucose. These results suggest that managing health behaviours and health factors to maintain high levels of cardiovascular health may be important in reducing the prevalence of infertility in female.

### 4.1 Comparison with previous studies

In our subgroup analysis, we observed that the upper limit of the confidence interval for the association between higher overall CVH scores, particularly for physical activity, BMI and blood glucose, and the odds of infertility was almost 1. This suggests that although there appears to be a trend towards lower odds of infertility with higher CVH scores, the association may not be as robust as reported. It’s important to interpret these findings with caution, given the potential for overestimation of the effect due to the proximity of the confidence interval to 1. This observation calls for further investigation into the specific components of CVH and their direct or indirect influence on female fertility outcomes.

To our knowledge, this is the first study to investigate the association of CVH levels assessed by LE8 with female infertility. Few previous studies have investigated whether there is an association between overall cardiovascular fitness levels and female infertility, and a recent Project Viva-based prospective study, also from the United States, investigated whether there was a difference in total CVH scores at midlife between those with and without a history of infertility in 468 female participants. The results showed that female with infertility had a 2.94-point lower CVH score compared with those without a history of infertility, which reinforces the reliability of our results in another way [26]. More studies have investigated the relationship between CVD and infertility, and these studies have found a variety of different CVD outcomes associated with infertility, although there are some differences in endpoint definitions from study to study [27]. Studies have shown that conditions like PCOS and endometriosis, often associated with infertility, are linked to a higher risk of cardiovascular diseases. female with these conditions are more likely to develop CVD risk factors such as hypertension and metabolic syndrome. This link suggests that infertility in female may be an indicator of broader health issues, including an increased risk of CVD [28]. A study focusing on the predictive value of female infertility for early cardiovascular disease found no increased risk of early CVD during a follow-up period. This suggests that the direct predictive value of infertility for CVD risk might not be as straightforward as previously thought [29]. There is evidence that cardiovascular risk factors present during childhood and adolescence, such as elevated LDL and total cholesterol, may be associated with adult subfertility [30]. This supports the notion that early-life cardiovascular health could influence reproductive outcomes. These findings collectively indicate a complex and multifaceted relationship between cardiovascular diseases and female infertility, emphasizing the need for a holistic approach to female’s health that considers both reproductive and cardiovascular aspects.

### 4.2 Potential biological mechanisms

In discussing the mechanisms underlying the negative association between CVH and female infertility, it is crucial to understand the multifaceted impact of the components of CVH on reproductive health. Physical activity emerges as a key factor, where regular exercise has been shown to enhance hormonal balance, reduce oxidative stress, and improve endothelial function, all of which are vital for maintaining regular ovulation and healthy reproductive organs [31, 32]. BMI plays another critical role, with high BMI often linked to hormonal imbalances and ovulatory dysfunction, conditions contributing to infertility. Obesity can lead to PCOS, a prevalent cause of infertility [33]. Moreover, blood glucose levels are intrinsically linked to reproductive health. Elevated blood glucose can lead to insulin resistance, a condition frequently associated with PCOS and subsequent infertility. This insulin resistance disrupts ovulation and adversely affects the quality of oocytes and embryos [34, 35]. Beyond these, other aspects of CVH also contribute to fertility. Poor dietary habits can lead to nutrient deficiencies affecting reproductive health [36]. Tobacco use is linked to reduced ovarian function and oocyte quality [19]. Inadequate sleep can disrupt hormonal rhythms, influencing fertility [37]. Dyslipidemia impacts hormone production and increases the risk of ovulatory dysfunction [38], and hypertension can impair uterine and ovarian blood flow [39]. These findings underscore a complex interplay between various components of CVH and female reproductive health, suggesting that the pathway to improving fertility might well lie in enhancing overall cardiovascular health.

### 4.3 Strengths and limitations

Our study’s strength primarily lies in leveraging the NHANES database, renowned for its sophisticated multi-stage, probability-based sampling method. This methodology enhances the representativeness of our findings across the diverse spectrum of the U.S. female population. Additionally, our comprehensive evaluation of cardiovascular health (CVH) through the Life’s Essential 8 (LE8) score provides an in-depth analysis of the multifaceted aspects of cardiovascular health and their potential implications on female infertility. Such an approach yields significant insights into how various components of CVH might influence reproductive outcomes. Despite these strengths, we acknowledge several limitations that merit consideration. The cross-sectional nature of our study inherently limits our capacity to establish causality from the observed associations between CVH components and female infertility. Although these associations are indicative of potential relationships, longitudinal studies are essential to confirm causation and elucidate the directional dynamics of these relationships. Moreover, while we have made efforts to adjust for a wide range of confounders, the potential for residual confounding exists. Given the intricate interplay between infertility and cardiovascular health, other unmeasured factors not accounted for in our study could influence the observed associations. This underscores the complexity of disentangling the web of factors impacting both infertility and CVH [40, 41]. A notable limitation is our reliance on self-reported data, particularly concerning infertility status and some CVH components. This reliance introduces the possibility of reporting bias, where the accuracy of self-reported information can vary, potentially impacting the validity of our conclusions. Future studies would benefit from incorporating more objective measures of infertility and CVH to mitigate this limitation [42, 43]. While our study capitalizes on the robust and nationally representative NHANES database to explore the association between cardiovascular health and female infertility, an important limitation to note is the selection of 3,969 female participants from an initial pool of 10,375. This selection was necessitated by the availability of complete data on infertility status and CVH components. We recognize that this reduction in sample size might influence the generalizability of our findings. Specifically, the exclusion of participants due to missing data could introduce selection bias, potentially affecting the representativeness of our study population relative to the broader U.S. female population. This limitation underscores the challenge of relying on self-reported data and the potential for non-response bias. Future research could benefit from strategies aimed at minimizing data missingness and employing multiple imputation techniques to better account for participants with incomplete data. Despite this limitation, our analysis provides valuable insights into the relationship between cardiovascular health and female infertility within the context of the available data. We remain cautious in interpreting our findings, acknowledging that the associations observed may not fully represent the underlying dynamics in the entire NHANES. Furthermore, our study’s focus on the U.S. population means that certain demographic and ethnic groups worldwide may not be adequately represented in our findings. Variations in lifestyle, access to healthcare, and prevalence of cardiovascular risk factors can impact the relationship between CVH and infertility in diverse populations. For instance, dietary habits, physical activity levels, and exposure to environmental pollutants, which are known to influence both CVH and reproductive health, vary widely across different societies.

## 5. Conclusion

Our study reveals a significant inverse association between cardiovascular health, as indicated by the LE8 score, and female infertility. This underscores the importance of maintaining optimal cardiovascular health for improving reproductive outcomes in female, highlighting a need for integrated health strategies that focus on both cardiovascular and reproductive wellness.

## Supporting information

S1 Table Definition and scoring approach for quantifying cardiovascular health, as per the American Heart Association’s Life’s Essential 8 score, and as applied in the National Health and Nutrition Examination Surveys. (PDF)

S2 Table Baseline characteristics of participants (age&lt;50 years) with CVH categories from LE8 score. (DOC)

S3 Table The associations between the Life’s Essential 8 cardiovascular health (CVH) score and infertility. (DOC)

S4 Table Subgroup analysis of the associations of Life’s Essential 8 cardiovascular health (CVH) score (per 10 scores) and infertility. (DOC)

## Abbreviations

BMI: Body mass index
CVD: Cardiovascular disease
CVH: Cardiovascular health
DASH: Dietary Approaches to Stop Hypertension
GAMs: Generalized additive models
HDL-C: high-density lipoprotein cholesterol
LE8: Life’s Essential 8
NCHS: National Center for Health Statistics
NHANES: National Health and Nutrition Examination Survey
PCOS: Polycystic ovary syndrome
PIR: Income-to-Poverty Ratio
