# Supplementary material for: Associations between cardiovascular health and female infertility: A national population-based study
Source: PLoS One. 2024 Jul 5;19(7):e0306476. doi: 10.1371/journal.pone.0306476 (PMC11226045; doi:10.1371/journal.pone.0306476)
Supplement: S2 Table — (DOC) [file pone.0306476.s002.doc]

**Table S2. Baseline characteristics of participants (age<50 years) with CVH categories from LE8 score**

| **Characteristics** | **Low (LE8 <50)** | **Moderate(50≤ LE8 <80 )** | **High (LE8 ≥80)** | ***P*-value** |
| --- | --- | --- | --- | --- |
| No. of participants in sample | 366 | 1,758 | 815 |  |
| Age, y (SD) | 38.37 ± 8.18 | 35.05 ± 8.54 | 33.03 ± 8.49 | <0.001 |
| Age of menarche, y (SD) | 12.22 ± 1.85 | 12.46 ± 1.75 | 12.90 ± 1.71 | <0.001 |
| PIR | 1.62 ± 1.28 | 2.26 ± 1.55 | 3.09 ± 1.65 |  |
| Race/ethnicity, n (%) |  |  |  | <0.001 |
| Mexican American | 51 (13.93%) | 325 (18.49%) | 109 (13.37%) |  |
| Others | 19 (5.19%) | 172 (9.78%) | 90 (11.04%) |  |
| Non-Hispanic White | 157 (42.90%) | 565 (32.14%) | 333 (40.86%) |  |
| Non-Hispanic Black | 105 (28.69%) | 431 (24.52%) | 83 (10.18%) |  |
| Other Hispanic | 34 (9.29%) | 265 (15.07%) | 200 (24.54%) |  |
| Marital status, n (%) |  |  |  | <0.001 |
| Married/Living with partner | 194 (53.01%) | 956 (54.38%) | 475 (58.28%) |  |
| Living alone | 172 (49.99%) | 802 (45.62%) | 340 (41.72%) |  |
| Education level, n (%) |  |  |  | <0.001 |
| Less than high school | 95 (25.96%) | 292 (16.61%) | 52 (6.38%) |  |
| High school | 101 (27.60%) | 357 (20.31%) | 108 (13.25%) |  |
| More than high school | 170 (46.44%) | 1,109 (63.08%) | 655 (80.37%) |  |
| Diabetes, n (%) |  |  |  | <0.001 |
| Yes | 102 (27.87%) | 172 (9.78%) | 18 (2.20%) |  |
| No | 251 (72.13%) | 1,586 (90.22%) | 797 (97.80%) |  |
| Pelvic infection disease, n (%) |  |  |  | <0.001 |
| Yes | 324 (88.52%) | 1657 (94.25%) | 799 (98.04%) |  |
| No | 42 (11.48%) | 101 (5.75%) | 16 (1.96%) |  |
| Infertility, n (%) |  |  |  | <0.001 |
| Yes | 298 (81.42%) | 1545 (87.88%) | 738 (90.55%) |  |
| No | 68 (18.58%) | 213 (12.12%) | 77 (9.45%) |  |
| AHA LE8 score (SD) |  |  |  |  |
| Mean total CVH score | 41.64 ± 6.75 | 65.83 ± 8.17 | 88.10 ± 5.42 | <0.001 |
| Mean DASH diet score | 19.89 ± 23.19 | 34.89 ± 29.92 | 63.43 ± 29.03 | <0.001 |
| Mean physical activity score | 10.41 ± 27.74 | 40.61 ± 46.05 | 88.32 ± 27.84 | <0.001 |
| Mean tobacco/nicotine exposure score | 36.19 ± 39.38 | 70.80 ± 38.26 | 91.42 ± 20.35 | <0.001 |
| Mean sleep health score | 65.36 ± 29.60 | 81.75 ± 25.02 | 91.14 ± 17.05 | <0.001 |
| Mean body mass index score | 21.67 ± 25.96 | 51.35 ± 35.64 | 85.60 ± 23.55 | <0.001 |
| Mean blood lipid score | 53.61 ± 30.83 | 76.22 ± 27.62 | 91.53 ± 18.13 | <0.001 |
| Mean blood glucose score | 69.26 ± 30.96 | 89.33 ± 20.86 | 98.75 ± 7.07 | <0.001 |
| Mean blood pressure score | 56.73 ± 30.42 | 81.66 ± 25.45 | 94.60 ± 14.29 | <0.001 |

Mean (SD) for continuous variables: the P value was calculated by the weighted linear regression model.

Percentages for categorical variables: the P value was calculated by the weighted chi-square test.

Cardiovascular health (CVH) is categorized into 3 grades,low:LE8 score <50, medium:50≤LE8 score <80, high:LE8 score ≥80.

Abbreviation: AHA, American Heart Association; LE8, Life’s Essential 8; CVH, cardiovascular health; DASH, Dietary Approaches to Stop Hypertension; PIR, The ration of family income to poverty.
