# Supplementary material for: Associations between cardiovascular health and female infertility: A national population-based study
Source: PLoS One. 2024 Jul 5;19(7):e0306476. doi: 10.1371/journal.pone.0306476 (PMC11226045; doi:10.1371/journal.pone.0306476)
Supplement: S3 Table — (DOC) [file pone.0306476.s003.doc]

Table S3. The associations between the Life’s Essential 8 Cardiovascular Health (CVH) score and infertility (age<50 years)

| **LE8/CVH** | **Model 1**  **[OR (95% CI)]** | **Model 2**  **[OR (95% CI)]** | **Model 3**  **[OR (95% CI)]** |
| --- | --- | --- | --- |
| **Total CVH score**  **(per 10 socres)** | 0.88 (0.83, 0.93) | 0.89 (0.84, 0.94) | 0.92 (0.88, 0.96) |
| CVH categories |  |  |  |
| Low (LE8 <50) | Ref. | Ref. | Ref. |
| Moderate (50≤ LE8 <80 ) | 0.78 (0.60, 0.98) | 0.84 (0.65, 1.05) | 0.78 (0.61, 0.97) |
| High (LE8 ≥80) | 0.65 (0.49, 0.86) | 0.73 (0.54, 0.98) | 0.61 (0.43, 0.86) |
| P for trend | <0.001 | <0.001 | <0.001 |
| **Subclass CVH scores**  **(per 10 socres)** |  |  |  |
| Mean DASH diet score | 0.96 (0.90, 1.01) | 0.97 (0.91, 1.02) | 0.98 (0.93, 1.02) |
| Mean physical activity score | 0.95 (0.91, 1.00) | 0.95 (0.90, 1.00) | 0.94 (0.90, 0.98) |
| Mean tobacco/nicotine exposure score | 0.93 (0.85, 1.00) | 0.94 (0.84, 1.02) | 0.94 (0.84, 1.02) |
| Mean sleep health score | 0.96 (0.92, 1.01) | 0.97 (0.93, 1.01) | 0.97 (0.94, 1.01) |
| Mean body mass index score | 0.93 (0.87, 0.98) | 0.94 (0.89, 0.99) | 0.94 (0.89, 0.99) |
| Mean blood lipid score | 0.97 (0.93, 1.01) | 0.97 (0.93, 1.02) | 0.98 (0.94, 1.02) |
| Mean blood glucose score | 0.93 (0.88, 0.98) | 0.91 (0.87, 0.99) | 0.92 (0.85, 0.99) |
| Mean blood pressure score | 0.96 (0.91, 1.02) | 0.96 (0.90, 1.02) | 0.95 (0.89, 1.02) |

Model 1 was unadjusted for covariates; Model 2 enhanced Model 1 by including age and ethnicity; and Model 3 further augmented Model 2 by integrating education level, family income-to-poverty ratio, age of menarche, pelvic infection disease, and diabetes status. Abbreviation: CVH, cardiovascular health; DASH, Dietary Approaches to Stop Hypertension.

^*^Tests for linear trends across three categories of cardiovascular health metrics scores were performed by modeling the median value within each category as a continuous variable
