# Supplementary material for: Associations between cardiovascular health and female infertility: A national population-based study
Source: PLoS One. 2024 Jul 5;19(7):e0306476. doi: 10.1371/journal.pone.0306476 (PMC11226045; doi:10.1371/journal.pone.0306476)
Supplement: S4 Table — (DOC) [file pone.0306476.s004.doc]

**Table S4.** Subgroup analysis of the associations of Life’s Essential 8 Cardiovascular Health (CVH) score (per 10 scores) and infertility (age<50 years)

| **Subgroup** | **OR (95%CI)** | **P for interaction** |
| --- | --- | --- |
| **Age** |  | 0.368 |
| < 30 years | 0.90 (0.81, 0.98) |  |
| ≥ 30 years | 0.92 (0.84, 1.00) |  |
| **Marital status** |  | 0.462 |
| Married/Living with partner | 0.95 (0.89, 1.00) |  |
| Living alone | 0.91 (0.85, 0.97) |  |
| **PIR** |  | 0.392 |
| < 1.3 | 0.89 (0.79, 0.99) |  |
| 1.3-3.5 | 0.94 (0.87, 1.01) |  |
| >3.5 | 0.93 (0.87, 1.01) |  |
| **Education level** |  | 0.421 |
| Less than high school | 0.84 (0.73, 0.95) |  |
| High school | 0.91 (0.84, 0.98) |  |
| More than high school | 0.91 (0.81, 0.99) |  |
| **Diabetes** |  | 0.333 |
| Yes | 0.85 (0.73, 0.97) |  |
| No | 0.93 (0.88, 0.97) |  |

Age, race, education level, family income-to-poverty ratio, age of menarche, pelvic infection disease, and diabetes status. Abbreviation: CVH, cardiovascular health; DASH, Dietary Approaches to Stop Hypertension.
